# Exclusive labeling of direct and indirect pathway neurons in the mouse neostriatum by an adeno-associated virus vector with Cre/lox system

**DOI:** 10.1016/j.xpro.2020.100230

**Published:** 2020-12-18

**Authors:** Shinichiro Okamoto, Kenta Yamauchi, Jaerin Sohn, Megumu Takahashi, Yoko Ishida, Takahiro Furuta, Masato Koike, Fumino Fujiyama, Hiroyuki Hioki

**Affiliations:** 1 Department of Cell Biology and Neuroscience, Juntendo University Graduate School of Medicine, Tokyo 113-8421, Japan; 2 Advanced Research Institute for Health Sciences, Juntendo University, Tokyo 113-8421, Japan; 3 Department of Neuroscience, Graduate School of Medicine, Kyoto University, Kyoto 606-8501, Japan; 4 Division of Cerebral Circuitry, National Institute for Physiological Sciences, Okazaki, Aichi 444-8787, Japan; 5 Department of Oral Anatomy and Neurobiology, Graduate School of Dentistry, Osaka University, Suita, Osaka 565-0871, Japan; 6 Laboratory of Histology and Cytology, Faculty of Medicine and Graduate School of Medicine, Hokkaido University, Sapporo, Hokkaido 060-8638, Japan

**Keywords:** Gene Expression, Microscopy, Neuroscience

## Abstract

We developed an adeno-associated virus (AAV) vector-based technique to label mouse neostriatal neurons comprising direct and indirect pathways with different fluorescent proteins and analyze their axonal projections. The AAV vector expresses GFP or RFP in the presence or absence of Cre recombinase and should be useful for labeling two cell populations exclusively dependent on its expression. Here, we describe the AAV vector design, stereotaxic injection of the AAV vector, and a highly sensitive immunoperoxidase method for axon visualization.

For complete details on the use and execution of this protocol, please refer to Okamoto et al. (2020).

## Highlights

• Exclusive labeling of two groups of neurons with different fluorescent proteins
• Detailed protocols from stereotaxic virus injection to immunostaining methods
• A signal enhancement method via peroxidase activity, BT-GO reaction
• Significant improvement in a signal-to-noise ratio by the cost-effective reaction

## Graphical Abstract

**Figure:**
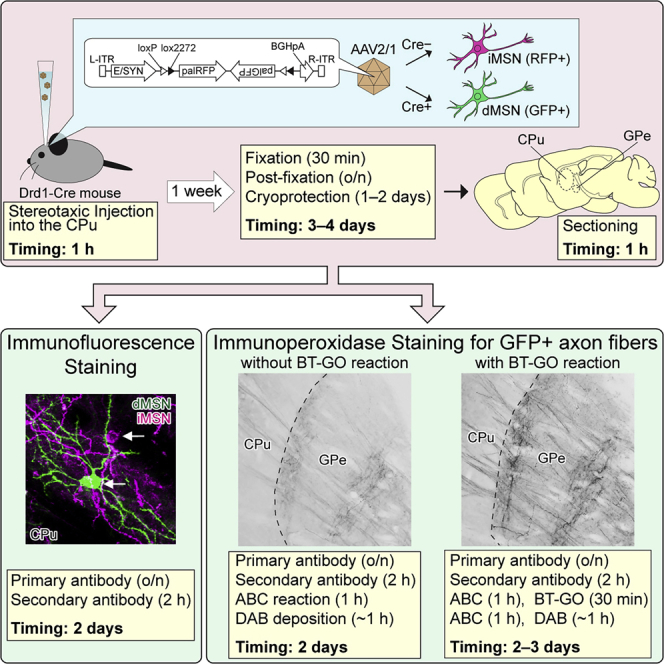

## Before you begin

**Figure 1:**
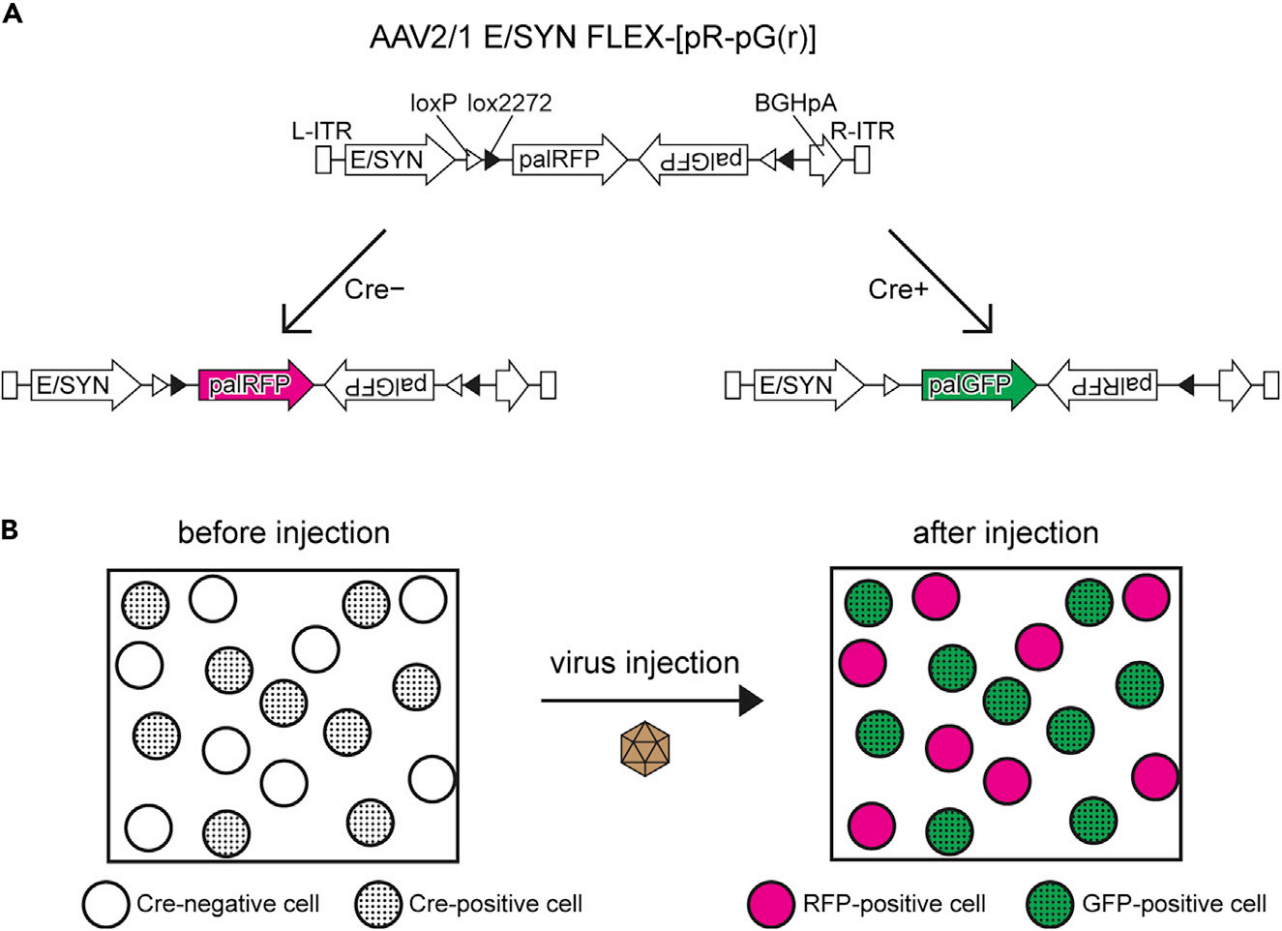
Exclusive labeling of Cre-positive and -negative cells by an AAV vector (A) Construct of the AAV vector. The vector expresses RFP or GFP in the absence or presence of Cre recombinase by a flip-excision (FLEX) switch. Both GFP and RFP are tagged with a membrane-targeting protein, palmitoylation signal (pal). (B) Schematic diagram of the exclusive labeling method. Two cell populations are selectively labeled with different fluorescent proteins based on the expression of Cre recombinase. BGHpA, a polyadenylation signal derived from bovine growth hormone gene; E/SYN, enhanced human synapsin I promoter (Hioki et al., 2007); ITR, inverted terminal repeat.

We developed a method using an adeno-associated virus (AAV) vector system to label two groups of neurons with different fluorescent proteins. The vector contains a flip-excision (FLEX) switch (Schnutgen et al., 2003), and expresses GFP or RFP based on the expression, or the lack, of Cre recombinase (Figures 1A and 1B). This labeling method offers a great advantage for the simultaneous labeling of mutually exclusive systems in the nervous system, such as excitatory and inhibitory neurons.

After preparation of the AAV vector, we injected the vector into the mouse neostriatum (caudate-putamen, CPu), and then analyzed the axonal projections of direct and indirect pathway medium-sized spiny neurons (dMSNs and iMSNs) (Okamoto et al., 2020). The production and purification methods of AAV vectors have been described in previous studies (Hamamoto et al., 2017; Ito et al., 2015; Iwano et al., 2018; Kataoka et al., 2014; Sohn et al., 2016; Sohn et al., 2017; Suzuki et al., 2015). We have also recently established a method to stably obtain a high-titer virus solution by recovering virus particles from cell pellets and the supernatant (medium) (Takahashi et al., 2020). For a detailed protocol of the AAV vector preparation, refer to the literature.

Here, we describe step-by-step procedures for stereotaxic injection of a virus solution into the mouse CPu, signal enhancement via peroxidase activity, and axon tracing. In particular, the signal enhancement method, namely the biotinylated tyramine-glucose oxidase (BT-GO) reaction, is cost-effective and straightforward (Furuta et al., 2009; Kuramoto et al., 2009) and should be useful for experiments that require specific staining with a high signal-to-noise ratio.

## Key resources table

**Table:** 

REAGENT or RESOURCE	SOURCE	IDENTIFIER
**Antibodies**		

Biotinylated goat antibody anti-rabbit IgG	Vector Laboratories	Cat# BA-1000; RRID: AB_2313606
Chicken polyclonal anti-GFP antibody	Aves Labs	Cat# GFP-1020; RRID: AB_10000240
Goat anti-rabbit IgG (H+L) highly cross-adsorbed secondary antibody, Alexa Fluor 568	Thermo Fisher Scientific	Cat# A-11036; RRID: AB_143157
Goat anti-chicken IgY (H+L) secondary antibody, Alexa Fluor 488	Thermo Fisher Scientific	Cat# A-11039; RRID: AB_2534096
Affinity-purified rabbit antibody against GFP	Tamamaki et al., 2000; Nakamura et al., 2008	N/A
Affinity-purified rabbit antibody against mRFP1	Hioki et al., 2010	N/A

**Bacterial and virus strains**		

AAV2/1-E/SYN-FLEX-[pR-pG(r)]	Okamoto et al., 2020	N/A

**Chemicals, peptides, and recombinant proteins**		

1,4-Diazabicyclo [2.2.2] octane (DABCO)	Nacalai tesque	Cat# 34811-72
Atipamezole hydrochloride (Antisedan)	Nihon Zenyaku Kogyo	N/A
Biotin–NHS	Calbiochem	Cat# 203112
Bovine serum albumin (BSA)	Nacalai tesque	Cat# 01863-77
Butorphanol tartrate (Vetorphale)	Meiji Seika Pharma	N/A
Diaminobenzidine-4HCl (DAB)	Dojindo	Cat# 347-00904
Dimethyl sulfoxide (DMSO)	Nacalai tesque	Cat# 13407-45
Di-sodium hydrogenphosphate (Na_2_HPO_4_)	Nacalai tesque	Cat# 31801-05
Di-sodium hydrogenphosphate 12-water (Na_2_HPO_4_･12H_2_O)	Nacalai tesque	Cat# 31722-45
Normal donkey serum	Sigma-Aldrich	Cat# S30-100ML
Formaldehyde solution	Nacalai tesque	Cat# 16223-55
Glucose oxidase (GO)	Nacalai tesque	Cat# 16831-14
Hydrochloric acid	Nacalai tesque	Cat# 18321-05
Hydrogen peroxide (H_2_O_2_) (31% w/v)	Santoku	N/A
Medetomidine hydrochloride (Domitor)	Nihon Zenyaku Kogyo	N/A
Midazolam (Midazolam Sandoz)	Sandoz	N/A
Monoethanolamine (2-aminoethanol)	Nacalai tesque	Cat# 23405-55
Sodium dihydrogenphosphate dihydrate (NaH_2_PO_4_･2H_2_O)	Nacalai tesque	Cat# 31718-15
Sodium hydroxide solution (NaOH) (10 mol/L)	Nacalai tesque	Cat# 94611-45
New MX	Matsunami Glass	Cat# FX00500
OCT compound	Sakura Finetek	Cat# 4583
Picric acid	Nacalai tesque	Cat# 27925-25
Phosphate buffered saline (PBS) (10×) (pH 7.4)	Nacalai tesque	Cat# 27575-31
Sodium azide	Nacalai tesque	Cat# 31233-55
Sodium pentobarbital (Somnopentyl)	Kyoritsu Seiyaku	N/A
Tris(hydroxymethyl)aminomethane (Tris)	Nacalai tesque	Cat# 35434-21
Triton X-100	Nacalai tesque	Cat# 35501-15
Tyramine hydrochloride	Sigma-Aldrich	Cat# T2879-1G
Xylene	Nacalai tesque	Cat# 36612-93
β-D-Glucose	Nacalai tesque	Cat# 16804-32
λ-Carrageenan	Wako Chemicals	Cat# 035-09693

**Critical commercial assays**		

VECTASTAIN Elite ABC Standard Kit	Vector Laboratories	Cat# PK-6100

**Experimental models: organisms/strains**		

Mouse: STOCK Tg (Drd1a-cre)150Gsat/Mmcd	MMRRC	RRID: MMRRC_029178-UCD

**Software and algorithms**		

ADOBE ILLUSTRATOR CS3	Adobe Systems	N/A

**Other**		

Aminopropyltriethoxysilane (APS)-coated glass slides	Matsunami Glass	Cat# APS-01
Auxiliary ear bar for mice	Narishige	EB-6
Bamboo tablet	Wacom Corporation	CTL-470/K0
Confocal laser scanning microscope TCS SP8	Leica Microsystems	N/A
D-700 camera	Nikon	N/A
Electro freeze	Yamato Koki	MC-802A
Fine-tip tweezers	Fine Science Tools	Dumont #5 Forceps
Glass capillary (diameter 2 mm)	Narishige	G-2
Hand drill	Minitor	Minimo
Low temperature circulator	EYELA	CCA-1112A
MICROPHOT-FXA	Nikon	N/A
Neo Sable round brush	Pentel	Cat# ZBNR-0
NESCO DERMARK R (a surgical marker-pen)	Alfresa Pharma	Cat# ND-2
Parafilm	Bemis Flexible Packaging	PM-996
Picospritzer III	Parker Hannifin Corporation	N/A
Puller	Narishige	PC-100
Sliding microtome	Leica Biosystems	SM2000R
Snow horn	Nippon Ekitan Corporation	N/A
Steel hole cutter (head diameter, 2.5 mm)	Minitor	BS1214
Stereomicroscope	Leica Microsystems	MZ16
Stereotaxic instruments for mice	Narishige	SR-5M-HT
Stereotaxic micromanipulator	Narishige	SM-15R
Digital slide scanner TOCO	CLARO	N/A

## Materials and equipment

**Table:** Fixative solution (pH 7.4)

Reagent	Final concentration	Amount
Saturated picric acid	75% (v/v)	750 mL
Na_2_HPO_4_	0.1 M	14.2 g
Formaldehyde solution	4% (v/v)	108 mL
NaOH	N/A	N/A
ddH_2_O	N/A	up to 1,000 mL

Adjust pH to 7.4 with NaOH.

Filter the solution through filter paper to remove any debris.

The solution may be preserved at 20°C–25°C for up to 1 year.

***Alternatives:*** 4% paraformaldehyde in 0.1 M PB can also be used with the fixative.

**Table:** PBS-X

Reagent	Final concentration	Amount
Triton X-100	0.3% (v/v)	1.5 mL
10× PBS	1×	50 mL
ddH_2_O	N/A	up to 500 mL

The solution may be preserved at 20°C–25°C for up to 2 weeks.

**Table:** 20% sodium azide solution

Reagent	Final concentration	Amount
Sodium azide	20% (w/v)	2 g
ddH_2_O	N/A	up to 10 mL

The solution may be preserved at 20°C–25°C for up to 1 year.

**Table:** PBS-XCD

Reagent	Final concentration	Amount
λ-carrageenan	0.12% (w/v)	120 mg
Normal donkey serum	1% (v/v)	1 mL
Triton X-100	0.3% (v/v)	0.3 mL
20% sodium azide solution	0.02% (v/v)	0.1 mL
10× PBS	1×	10 mL
ddH_2_O	N/A	up to 100 mL

The solution may be preserved at 4°C for up to 1 year.

### Biotinylated tyramine (BT) solution

**Figure 2:**
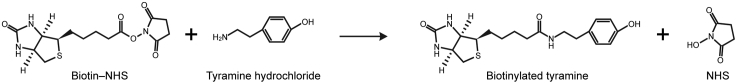
Chemical formula to produce biotin-tyramine

Prepare BT solution as follows (Figure 2):

- Dissolve 3.5 mg of biotin–NHS in 36.5 μL of DMSO.
- Dissolve 15 mg of tyramine hydrochloride in 300 μL of DMSO (in the next step, use only 36.5 μL out of 300 μL).
- Mix well 36.5 μL of biotin–NHS solution and 36.5 μL of tyramine hydrochloride solution by inversion.
- Incubate the mixture for 12–24 h at 20°C–25°C with rotation and protection from light.
- Add 7.3 μL of monoethanolamine to the mixture.
- Incubate it for 4 h at 20°C–25°C to inactivate the remaining free biotin–NHS with rotation and protection from light.
- Keep the BT solution at 4°C for up to 2 months. For a more extended duration up to 3 years, store the solution at −80°C.

***Note:*** When all biotin–NHS reacts with tyramine hydrochloride, 128 mM of BT solution will be obtained.

**Table:** 0.2 M phosphate buffer (PB) (pH 7.4)

Reagent	Final concentration	Amount
NaH_2_PO_4_･2H_2_O	0.04 M	11.8 g
Na_2_HPO_4_･12H_2_O	0.16 M	116 g
ddH_2_O	N/A	up to 2 L

The solution may be preserved at 4°C for up to 1 year.

**Table:** Glucose oxidase (GO) solution

Reagent	Final concentration	Amount
Glucose oxidase	1 mg/mL	1 mg
0.2 M PB	0.1 M	0.5 mL
ddH_2_O	N/A	0.5 mL

Dispense to 100 μL each and store at −80°C. The solution may be preserved at 4°C for up to 1 month.

**Table:** ß-D-glucose solution

Reagent	Final concentration	Amount
ß-D-glucose	200 mg/mL	200 mg
ddH_2_O	N/A	1 mL

Dispense to 100 μL each and store at −80°C. The solution may be preserved at 4°C for up to 1 month.

**Table:** Mounting solution for fluorescence microscopy

Reagent	Final concentration	Amount
Glycerol	50% (v/v)	10 mL
DABCO	2.5% (w/v)	0.5 g
20% sodium azide solution	0.2% (w/v)	20 μL
10× PBS	1×	2 mL
ddH_2_O	N/A	up to 20 mL

The solution may be preserved at 4°C for up to 1 year.

**Table:** 0.5 M Tris-HCl (pH 7.6)

Reagent	Final concentration	Amount
Tris	0.5 M	60.57 g
ddH_2_O	N/A	up to 1 L

Adjust pH to 7.6 with HCl.

Before use, dilute the solution 10-fold with ddH_2_O (50 mM Tris-HCl).

The solution may be preserved at 20°C–25°C for up to 1 year.

## Step-by-step method details

### Virus injection

**Timing: ~1 h**

Here, we describe the stereotaxic injection of an AAV vector solution (200 nL) into the mouse CPu.

**Figure 3:**
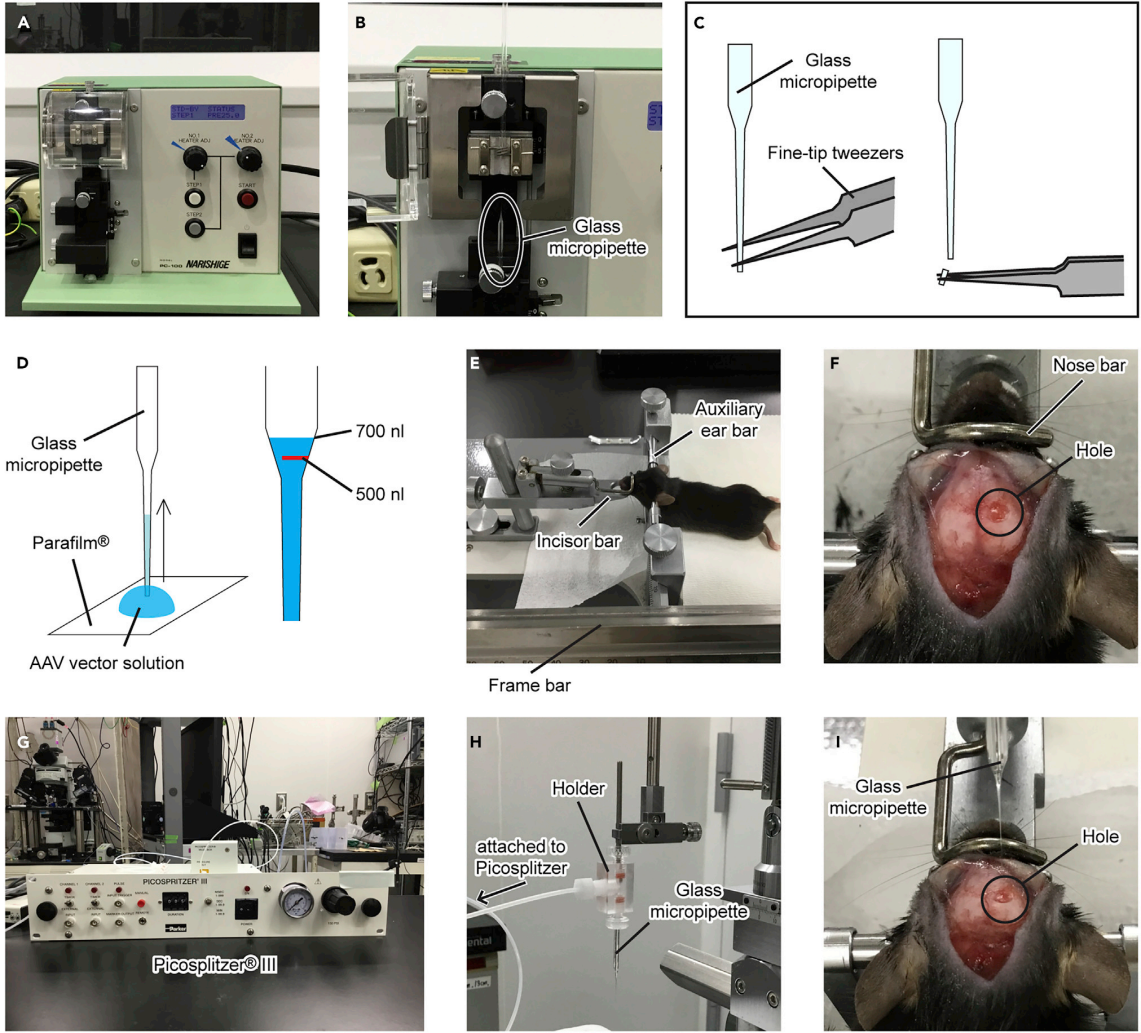
Injection of the AAV vector solution (A) PC-100 puller. (B) Fabrication of a glass micropipette. (C) Folding the tip of a glass micropipette (D) Aspiration of the AAV vector solution into the glass micropipette. First, 500 nL of the solution is aspirated to mark the top surface with a permanent marker, and then another 200 nL is aspirated. (E) Head fixation of an anesthetized mouse with a stereotaxic instrument. (F) Perforation with a hand drill. (G) Picospritzer III connected to a high-pressure N_2_ cylinder. (H) Attaching the glass micropipette to the holder of Picospritzer III. (I) Inserting the micropipette into the brain.

***Note:*** The setting values will vary depending on the filament. Find the best conditions for your purpose.

1. Disinfect the instruments for use in the injection experiment with 70% ethanol. Please refer to institutional guidelines for specific instructions on proper aseptic technique.
2. Fabricate a glass micropipette using a PC-100 puller with a single pulling mode. Set the heating value to 73.5 and pulling force to 125 g (Figures 3A and 3B).

3. Fold the tip with fine-tip tweezers and check under a microscope that the tip is 50–70 μm in diameter (Figure 3C).
4. Dilute AAV2/1-E/SYN-FLEX-[pR-pG(r)] vector solution with PBS containing 2% (w/v) BSA to 1 × 10^11^ infectious unit/mL.
5. Place the diluted solution on ice.

***Note:*** The solution may be preserved at 4°C for up to 1 month.

6. Aspirate 500 nL of an AAV vector solution on a Parafilm sheet through the tip of a glass micropipette using capillary action, and mark the top surface of the solution with a permanent marker. Then, aspirate 200 nL of a virus solution again (700 nL in total).

**CRITICAL:** When injecting deep into the brain, the top surface of the solution becomes invisible, making it difficult to ascertain the injection volume. Aspirate the extra solution (e.g., 500 nL) and mark the level with a permanent marker so that you can inject the exact amount of the virus solution (Figure 3D).

7. Anesthetize a Drd1-Cre BAC transgenic mouse (8–16 weeks) by intraperitoneal injection of a mixture of medetomidine (Domitor; 0.3 mg/kg), midazolam (Midazolam Sandoz; 4 mg/kg), and butorphanol tartrate (Vetorphale; 5 mg/kg) in saline (0.9% NaCl). Please refer to institutional guidelines for proper anesthesia.
8. Mount the anesthetized mouse into a stereotaxic instrument using an auxiliary ear bar. Adjust the position of the mouth and nose fixing clamp (incisor bar) until the lambda and bregma are equal in height (flat-skull position) (Figure 3E).
9. Make an incision in the scalp with a scalpel along the midline to expose the skull.
10. After marking the skull above the CPu with a surgical marker-pen (NESCO DERMARK R), thin the skull above the marked site using a hand drill with a steel hole cutter (Figure 3F). The injection coordinates for the CPu are as follows: 0.8 mm anterior to bregma, 2.0 mm lateral to the midline, and 2.5 mm ventral to the brain surface.

**CRITICAL:** While thinning the skull by a hand drill, wet with saline appropriately to avoid damage to the cortices by frictional heat.

11. Carefully remove the remaining bone using fine-tip tweezers to expose the surface of the brain.
12. Set the Picospritzer III to 40 psi injection pressure and 5 ms injection duration (Figures 3G and 3H).

**CRITICAL:** Eject a drop of virus solution from the tip of the glass micropipette to make sure it is not clogged, before inserting it into the brain. Troubleshooting 1

13. Move the glass micropipette to the brain surface and slowly insert it into the target (Figure 3I). Pull the micropipette upward approximately 0.05 mm to make space for the virus solution to diffuse. Troubleshooting 2, 3, and 4
14. Inject the virus solution into the mouse CPu by pressure pulses through a glass micropipette attached to Picospritzer III for 5 min. Troubleshooting 5
15. Leave the micropipette in place for 5 min, and then remove it slowly.
16. After closing and sterilizing the wound, administer antibiotics (e.g., gentamicin ointment) locally.
17. Recover the mouse from anesthesia by intraperitoneal injection of atipamezole (Antisedan; 1.5 mg/kg) in saline after approximately 15 min. Please refer to institutional guidelines for anesthesia management.
18. Maintain the mouse in specific pathogen-free conditions under a 12 h light/dark cycle with *ad libitum* access to food and water for 1 week after the AAV injection.

### Tissue preparation

**Timing: 2–3 days**

***Note:*** Filtrate PBS and the fixative solution through filter paper before perfusion.

Here, we introduce the preparation of sections for free-floating immunostaining.

19. Anesthetize the mouse deeply by intraperitoneal injection of sodium pentobarbital (Somnopentyl; 200 mg/kg) in saline. Please refer to institutional guidelines for proper anesthesia.
20. Open the thoracic cavity with surgical scissors, cut the right atrial appendage to bleed, and immediately insert a 22-gauge needle from the apex into the left ventricle of the heart.
21. Perfuse with 20 mL of PBS using a syringe for 3 min to remove the blood from the circulatory system.
22. Perfuse with 20 mL of a fixative solution using another syringe at the same speed.

**CRITICAL:** Be careful not to allow any small bubbles to enter during the perfusion. The inclusion of bubbles results in incomplete fixation.

**Figure 4:**
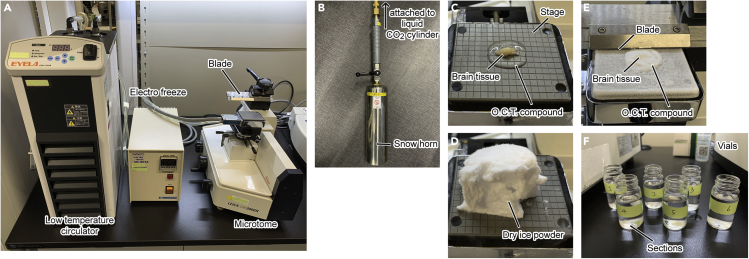
Section preparation (A) Sliding microtome equipped with a freezing device. (B) Equipment to make dry ice powder (snow horn). (C) Mounting brain tissue onto the stage with OCT compound. (D) Freezing brain tissue with dry ice powder. (E) Cutting brain tissue sections on the sliding microtome. (F) Brain sections serially collected in 6 vials.
26. Mount the brain tissue onto the stage of a freezing microtome with OCT compound, and adjust the stage angle to keep the sagittal plane of the brain horizontal (Figure 4C).
27. Turn on the electro-freezing component and set it to −25°C.
28. Quickly freeze the brain tissue using dry ice powder and leave it in place for at least 5 min (Figure 4D).

***Note:*** We recommend dry ice powder prepared by a snow horn for rapid tissue freezing (Figure 4B).

23. Remove the brain from the skull and place it in the same fixative solution for 12–24 h at 4°C.
24. Replace the fixative solution with 30% (w/v) sucrose in 0.1 M PB and gently shake it for 1–2 days at 4°C to cryoprotect the brain tissue.
25. Approximately 30 min before sectioning, turn on the low temperature circulator and set it to 4°C (Figure 4A).

29. Cut the brain tissue into 20-μm-thick parasagittal sections (Figure 4E), collect the sections with a paintbrush, and store them in 6 vials containing 0.02% sodium azide in 0.1 M PB (Figure 4F). Each vial will contain around 20 sections.

***Note:*** For long-term storage (e.g., 5 years), increase the concentration of sodium azide to 0.2%.

### Immunofluorescence staining

**Timing: 2 days**

Here, we explain the procedure for double immunofluorescence staining for GFP and RFP. All the incubations are performed at 20°C–25°C with a gentle shake.

30. Wash the sections with 8 mL of PBS for 10 min twice, and then with PBS-X for 30 min.
31. Incubate the sections for 12–24 h with a mixture of 20 μg/mL of chicken polyclonal anti-GFP antibody and 1 μg/mL of affinity-purified rabbit antibody against mRFP1 in 500 μL of PBS-XCD.
32. Wash the sections with 8 mL of PBS-X for 10 min twice.
33. Incubate the sections for 2 h with a mixture of 5 μg/mL of AlexaFluor (AF) 488-conjugated antibody against chicken IgY and 5 μg/mL of AF568-conjugated antibody against rabbit IgG in 500 μL of PBS-XCD.
34. Wash the sections with 8 mL of PBS-X for 20 min twice, and then with 8 mL of PBS for 20 min twice.
35. Mount the sections onto APS-coated glass slides.
36. Apply a coverslip with a mounting medium for fluorescence microscopy.

***Note:*** The sections can be kept for about 2 years at −20°C.

**Figure 5:**
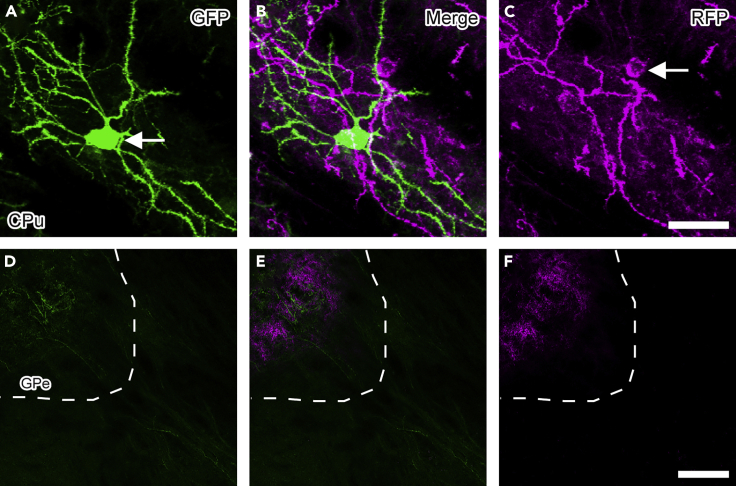
Exclusive labeling of dMSNs and iMSNs (A–C) The presence of spines on the dendrites indicates that GFP-positive and RFP-positive cells are dMSNs and iMSNs, respectively. Arrows indicate the cell bodies. Scale bar, 30 μm. (D–F) In the GPe, GFP-positive fibers were sparsely distributed, whereas RFP-positive fibers were dense. GFP-positive, but not RFP-positive, fibers extend caudally beyond the GPe, forming the direct pathway to the output nuclei of the basal ganglia. Scale bar, 100 μm.

37. Observe the sections under a confocal laser scanning microscope (Figures 5A–5F). Troubleshooting 6 and 7

### Immunoperoxidase staining

**Timing: 4 days**

**CRITICAL:** Before incubation with the sections, mix solutions A and B of ABC Elite kit at 1:100 dilution each in 500 μL of PBS-X and incubate the mixture for at least 30 min at 20°C–25°C with a gentle shake to form the avidin-biotinylated peroxidase complex (ABC).

We developed a cost-effective and straightforward signal amplification method called BT-GO reaction. Here, we describe the protocol in detail. All the incubations are performed at 20°C–25°C with a gentle shake.

38. Wash the sections with 8 mL of PBS for 10 min twice.
39. Incubate the sections for 30 min with 8 mL of 1% (v/v) H_2_O_2_ in PBS to inactivate endogenous peroxidase activity.
40. Wash the sections with 8 mL of PBS-X for 10 min twice.
41. Incubate the sections for 12–24 h with 0.1 μg/mL of affinity-purified rabbit antibody against GFP or mRFP1 in 500 μL of PBS-XCD.
42. Wash the sections with 8 mL of PBS-X for 10 min twice.
43. Incubate the sections for 2 h with 10 μg/mL biotinylated goat antibody against rabbit IgG in 500 μL of PBS-XCD.
44. Wash the sections with 8 mL of PBS-X for 10 min twice.
45. Incubate the sections for 1 h with 1:100-diluted avidin-biotinylated peroxidase complex (ABC) in 500 μL of PBS-X.
46. Wash the sections with 8 mL of PBS-X for 10 min twice.
47. Wash the sections with 8 mL of 0.1 M PB for 10 min twice.
48. Incubate the sections for 5 min with a BT-GO reaction mixture containing 1:50,000-diluted BT solution, 3 μg/mL of GO, and 1% BSA in 1 mL of 0.1 M PB.

***Note:*** The optimal concentration of the BT solution used in the reaction should be determined by the end user. We recommend a dilution of the solution from 1:5,000 to 1:500,000 for the trial.

49. Add 10 μL of β-D-glucose solution to a final concentration of 2 mg/mL and mix the solution and sections well.
50. Incubate the sections for 30 min (Figure 6A).

**Figure 6:**
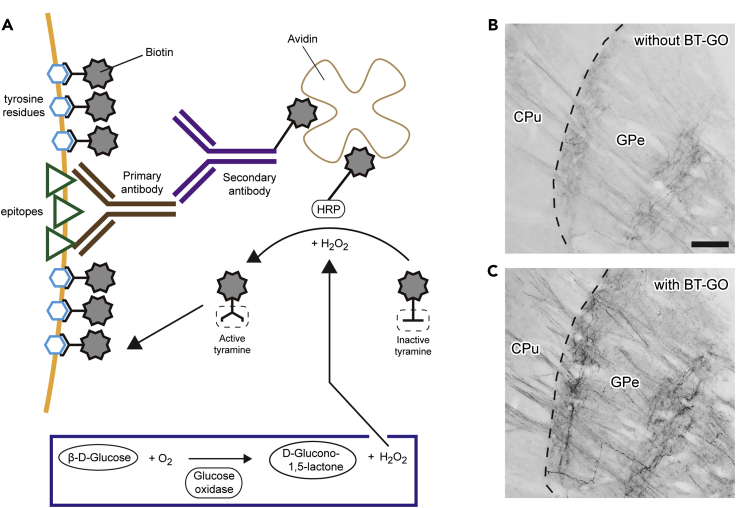
Signal enhancement with the BT-GO reaction (A) Schematic diagram of the signal amplification method with the BT-GO reaction. BT is deposited on tyrosine residues in the tissue via the peroxidase activity of ABC. This deposition requires H_2_O_2_, and the chemical reaction between glucose and GO supplies H_2_O_2_ stably. (B and C) Visualization of GFP-labeled dMSN axon fibers in the GPe without (B) or with (C) the BT-GO reaction. While DAB deposition through the peroxidase activity of ABC gave a weak signal (B), the BT-GO reaction markedly amplified the signals of the axon fibers. (C) is the adjacent section of (B). Scale bar, 100 μm.
51. Wash the sections with 8 mL of 0.1 M PB for 10 min.
52. Wash the sections with 8 mL of PBS-X for 10 min twice.
53. Incubate the sections for 1 h with 1/100-diluted ABC in 500 μL of PBS-X.
54. Wash the sections with 8 mL of PBS-X for 10 min twice.
55. Wash the sections with 8 mL of PBS for 10 min twice.
56. Incubate the sections for 30–60 min with 0.02% (w/v) DAB and 0.0001% (v/v) H_2_O_2_ in 8 mL of 50 mM Tris-HCl (pH 7.6).

**CRITICAL:** Check the progress of DAB deposition under the microscope and stop the reaction at a high signal-to-noise ratio. If DAB deposition is weak and slow, additional H_2_O_2_ can be added directly to the reaction solution.

57. Wash the sections with 8 mL of PBS for 10 min three times.
58. Mount all the stained sections serially onto APS-coated glass slides.
59. Dry up the sections.
60. Soak the glass slides in ethanol-water mixtures of 30, 50, and 70% (v/v) for 30 min each, and dip the slides into 95% (v/v) ethanol in water.
61. Soak the glass slides in 100% ethanol for 12–24 h.
62. Dip the glass slides into a xylene-ethanol mixture of 50% (v/v) and soak the slides in 100% xylene for 12–24 h.
63. Coverslip the sections with a mounting medium for bright-field microscopy, NEW MX.
64. Observe the sections under a bright-field microscope, MICROPHOT-FXA, (Figures 6B and 6C) and capture images using a Nikon D-700 camera. Troubleshooting 8

***Note:*** Specimens can be stored for at least 10 years at 20°C–25°C with protection from light.

### Axon tracing

**Timing: ~4 days**

By using the virtual slide system, the efficiency of the two-dimensional reconstruction is greatly increased. We briefly describe the reconstruction method using a TOCO virtual slide scanner.

65. Acquire a color image of the entire section with a spatial resolution of 1.038 μm/pixel using the digital slide scanner TOCO with a 10× objective lens.
66. Trace and digitize the axons of DAB-stained neurons on the images with a digital pen tablet and a graphic software ADOBE ILLUSTRATOR CS3.

***Alternatives:*** Virtual slide systems from other manufacturers are also available.

***Note:*** Axons are sometimes out of focus, where multiple axon fibers cross one another. Additional observations with a bright field microscope may also be required to reconstruct the axons accurately.

## Expected outcomes

The stereotaxic injection method allows us to deliver the transgene to the restricted area of the mouse CPu (Figure 3). The range of the infection area will be isotropic, with a radius of 0.5 to 0.8 mm. If the infection area is further confined, it may be more effective to use iontophoresis than pressure injection (Oh et al., 2014; Wang et al., 2014). Moreover, section preparation with a freezing microtome not only completes the process in a short time but may also lead to the improvement of antibody penetration and a signal-to-noise ratio due to freeze-thaw treatment (Figure 4).

By injecting an AAV vector solution into the CPu of a Drd1-Cre mouse, we successfully visualized dMSNs and iMSNs with different fluorescent proteins, GFP and RFP. While the projection of RFP-positive axon fibers was restricted to the external segment of the globus pallidus (GPe), GFP-positive axon fibers extended caudally beyond the region (Figure 5). We then amplified the signals of GFP-positive and RFP-positive axon fibers in the GPe using the BT-GO reaction (Figure 6). The reaction remarkably enhanced the signals while leaving the noise unaltered, thus contributing to the increase in the signal-to-noise ratio.

## Limitations

The present exclusive labeling method allows us to visualize two cell populations with different fluorescent proteins based on the expression of Cre recombinase. However, the Cre-negative population may contain multiple cell types. Indeed, in addition to iMSNs, interneurons in the CPu not expressing Cre recombinase were labeled with RFP in the present study. Nonetheless, they can be distinguished by their morphology, such as the cell body size and the presence of spines on the dendrites (Okamoto et al., 2020). Given that interneurons do not send axons outside of the CPu, the distributions of dMSN and iMSN axon fibers, which are the focus of our study, can be quantitatively analyzed within the GPe. When using this exclusive labeling method, careful attention should be paid to the characteristics of the Cre-negative cell population.

Direct delivery of the virus vectors with stereotaxic injections has the advantage of being suitable for any specific region of the animal brain at any time. On the other hand, a disadvantage is that the infection area is limited. When a wide-area gene transfer is required, an AAV serotype that efficiently crosses the blood-brain barrier, such as AAV-PHP.eB (Chan et al., 2017), should be administrated intravenously.

The BT-GO reaction efficiently increases the signal-to-noise ratio; we (Furuta et al., 2009; Kuramoto et al., 2009) and others (Igarashi et al., 2012; Sun et al., 2020) have applied this method to axonal projection analysis. However, bright-field staining by DAB deposition via the BT-GO reaction has the drawback that the GFP and RFP signals cannot be observed simultaneously in the same section. The development of signal amplification methods that enable fluorescent multi-color labeling is expected in future studies.

## Troubleshooting

### Problem 1

No virus solution is released from the glass micropipette (step 12).

### Potential solution

Set the injection duration of Picospritzer III to a longer time. If the solution is still not ejected, check the shape of the glass micropipette tip under a stereomicroscope.

### Problem 2

The glass micropipette cannot be inserted into the brain (step 13).

### Potential solution

Perform a small incision in the dura mater with fine-tip tweezers or a needle.

### Problem 3

The infection site is out of the target position (step 13).

### Potential solution

In stereotactic injection, it is critical to mount animals into a stereotaxic apparatus properly. In particular, ensure that the ear bars are inserted properly.

### Problem 4

Infected cells are frequently observed in the area of passage of the glass micropipette (step 13).

### Potential solution

The following points should be verified: 1) the glass micropipette is thin, 2) there is no bubble inside the glass micropipette, 3) slow injection of the virus solution, and 4) slow removal of the glass micropipette.

### Problem 5

Excessive numbers of cells are labeled with the virus vectors (step 14).

### Potential solution

The concentration of the solution should be determined according to the purpose of the experiment. Lower concentrations label fewer neurons.

### Problem 6

The expression of fluorescent proteins is low (step 37).

### Potential solution

The survival time of animals after virus injection should be determined based on the purpose of the experiments. In general, longer survival times result in higher expression.

### Problem 7

The fluorescent signal rapidly fades (step 37).

### Potential solution

It is recommended to evaporate as much water as possible in the mounting medium for fluorescence microscopy.

### Problem 8

High background immunostaining (step 64).

### Potential solution

Higher concentrations of primary antibodies may result in higher background immunostaining, especially in immunoperoxidase staining. First, the concentration of the primary antibody and then the concentration of the BT solution should be reduced (primary antibodies, a factor of 5; BT solution, a factor of 10–100).

## Resource availability

### Lead contact

Further information and requests for resources and reagents should be directed to and will be fulfilled by the Lead Contact, Hiroyuki Hioki (h-hioki@juntendo.ac.jp).

### Materials availability

All plasmids used in this study are available from the Lead Contact upon request.

### Data and code availability

This study did not generate any new data or code.
